# Serum valeric acid stimulates lung epithelial cilia assembly and improves prognosis in patients with severe respiratory infections

**DOI:** 10.3389/fphar.2025.1761517

**Published:** 2026-01-12

**Authors:** Yabin Zhang, Yuqing Zhang, Lin Wang, Beibei Liu, Jiajia Zheng, Jiabao Cao, Lixin Xie, Ning Shen, Jun Wang

**Affiliations:** 1 Shandong First Medical University and Shandong Academy of Medical Sciences, Jinan, China; 2 CAS Key Laboratory of Pathogenic Microbiology and Immunology, Institute of Microbiology, Chinese Academy of Sciences, Beijing, China; 3 University of Chinese Academy of Sciences, Beijing, China; 4 School of Medicine, Nankai University, Tianjin, China; 5 College of Pulmonary and Critical Care Medicine, 8th Medical Center of Chinese PLA General Hospital, Beijing, China; 6 Department of Pulmonary and Critical Care Medicine, Peking University Third Hospital, Beijing, China; 7 Department of Laboratory Medicine, Peking University Third Hospital, Beijing, China

**Keywords:** cilia, metabolomics, severe pneumonia, valeric acid, Wnt pathway

## Abstract

**Introduction:**

Globally, respiratory infections remain a leading cause of mortality, with treatment efficacy increasingly challenged by antimicrobial resistance. This study aimed to investigate the role of serum metabolites in the prognosis of severe human pneumonia.

**Methods:**

Untargeted and targeted serum metabolomics were performed on intensive care unit (ICU) patients. Experimental validation was conducted in a murine bacterial infection model and cellular models. RNA sequencing was used for mechanistic exploration to identify the signaling pathways regulated by the key metabolite.

**Results:**

Valeric acid, a short-chain fatty acid, was significantly elevated in survivors compared with non-survivors of severe pneumonia. In the murine *Klebsiella pneumoniae* model, valeric acid treatment alleviated infection severity, reduced body weight loss, lung inflammation, and bacterial load. Mechanistically, RNA sequencing revealed that valeric acid suppresses IL-17-associated inflammation and upregulates pathways related to mucociliary clearance. We further delineated the underlying mechanism, finding that valeric acid acts as a histone deacetylase (HDAC) inhibitor, specifically targeting HDAC3. This inhibition activates the canonical Wnt/β-catenin signaling pathway, leading to the upregulation of the master transcriptional regulator Foxj1 and subsequent promotion of cilia assembly and function in airway epithelia.

**Discussion:**

The findings establish a protective role for the gut microbiome-derived valeric acid in respiratory infections via the novel HDAC-Wnt-FOXJ1 axis, revealing its potential as a therapeutic agent to improve clinical outcomes.

## 1 Introduction

Respiratory infections remain a leading cause of death and pose a serious threat to human health. Seasonal flu and other viral infections, community-acquired pneumonia (CAP) and hospital-acquired pneumonia (HAP) represent the most common forms of respiratory infections requiring clinical treatment and hospitalization (Magill et al., 2014; Zaragoza et al., 2020); and fast evolution of drug resistance in the common pathogens continues to challenge the efficacy of treatment and aggravate the health burden. Besides these common bacterial and viral infections, emerging new pathogens such as Middle East respiratory syndrome coronavirus, SARS-CoV, and SARS-CoV-2 also lead to additional burdens of respiratory infections and in the case of SARS-CoV-2, the most recent global pandemic of COVID-19 (Algaissi et al., 2020; Schwartz, 2020; Li et al., 2021; May, 2021). Due to the ever-increasing threat and burdens, a large number of investigations have revealed important anthropometric factors associated with clinical prognosis, in particular, patient age, gender, body-mass index; genetic factors including blood types and HLAs, among others (Lin et al., 2003; Dai, 2020; Ellinghaus et al., 2020; Bouziotis et al., 2022).

It is also increasingly accepted that the complex microbial communities in human, also termed commensal microbiome, play important roles in shaping outcome of lung infection. The respiratory tract microbiome is usually considered to be relatively low in biomass, compared to gastrointestinal tract. Nonetheless, the commensal fungi, bacteria, and viruses have been shown to be required for maintaining the immune balances of the lung environment as well as the barrier functions; and in case of infection, opportunistic pathogens in the microbiome can promote further inflammation and/or incur secondary infections and worsen the prognosis. Gut microbiome can also exert influences onto the respiratory system via the gut-lung axis, in which the gut microbiome modulate infection responses via its metabolites such as deaminotyrosine that reduces inflammation in mice model of influenza infection, and stimulate the production of cytokines such as GM-CSF (Steed et al., 2017). Moreover, it has been reported that the gut microbiome metabolite acetic acid can protect against infection by the respiratory syncytial virus and the influenza A virus (Antunes et al., 2019; Niu et al., 2023). Therefore, regulating the composition of the gut microbiome and its metabolites helps enhance pulmonary immunity and defend against respiratory infections.

In our study, we analyzed a cohort of severe HAP or CAP patients and compared the serum metabolome according to clinical outcomes, identifying a gut microbiome-derived short-chain fatty acid (SCFA), valeric acid, whose concentration was positively associated with survival rate. We experimentally validated the protective effect of valeric acid in mouse models of lung infection by bacterial pathogens, an effect that depended on its ability to reduce IL-17-associated inflammation and upregulation of cilia assembly related pathways. Functionally, valeric acid inhibited HDAC activity and upregulated Wnt-FOXJ1 pathway to promote cilia assembly. Hence, we identify a new microbial-derived molecule with general protective effect against respiratory infection and pave the way for improved intervention and treatment.

## 2 Materials and methods

### 2.1 Patient enrollment and sample collection

This study enrolled patients with severe pneumonia who were admitted to the ICU of Peking University Third Hospital and the First Medical Center of Chinese People’s Liberation Army General Hospital between July 2019 and January 2020. The inclusion criteria were as follows: (1) aged ≥16 years; (2) admitted to the ICU; and (3) clinically diagnosed with severe pneumonia. The exclusion criteria included: (1) pregnant or lactating women; (2) patients receiving immunosuppressive therapy; and (3) individuals unable to provide valid informed consent. Based on these inclusion and exclusion criteria, 57 patients were finally enrolled in this study. These patients were further divided into two groups (survivors and non-survivors) according to their clinical outcomes.

Serum samples were collected from patients and stored at −80 °C for long-term storage. All participants were informed about this study and obtained written informed consent before participation. This study was approved by the Ethics Committee of Peking University Third Hospital and the First Medical Center of the Chinese People’s Liberation Army General Hospital, and strictly adhered to the principles of the Declaration of Helsinki.

### 2.2 Bacterial culture

*Klebsiella pneumoniae* (*K. pneumoniae*) strain ATCC 700603 was cultured on LB agar plates and incubated overnight at 37 °C. A single colony was then inoculated into LB broth and incubated overnight at 37 °C with shaking at 200 rpm. When the bacteria reached the mid-logarithmic growth phase, the culture was harvested, and the bacterial cells were collected by centrifugation at 10,000×g for 10 min at 4 °C, followed by resuspension in phosphate-buffered saline (PBS).

### 2.3 Mouse experiments

Female C57BL/6 mice (6–8 weeks old) were purchased from GemPharmatech Co., Ltd. (Jiangsu, China) and maintained in-house for 1 week before any treatment. All mice were housed in a specific pathogen-free and biosafety level 2 laboratory facility, maintained on a 12 h light-dark cycle, and had *ad libitum* access to sterile water and standard rodent chow. Age-matched mice were used for all experiments, and they were randomly assigned to different experimental groups. All animal experiments were approved by the Animal Ethics Committee of the Institute of Microbiology, Chinese Academy of Sciences. All animal experiments were conducted under isoflurane anesthesia, and every effort was made to minimize suffering.

To evaluate the *in vivo* effects of valeric acid under non-infectious conditions, mice were randomly assigned to PBS and valeric acid groups. Mice in the valeric acid group received an intraperitoneal (i.p.) injection of 500 μL 50 mM valeric acid, whereas mice in the PBS group were administered 500 μL PBS. After 24 h of treatment, all animals were euthanized, and serum and lung tissues were collected for further analysis.

To assess the effects of valeric acid under infectious conditions, mice were randomly divided into three groups, namely uninfected treated with PBS, *K. pneumoniae*-infected treated with PBS, and *K. pneumoniae*-infected treated with valeric acid. Briefly, mice were anesthetized with 5% isoflurane (oxygen flow rate, 0.5 L/min) and maintained with 1.5% isoflurane at room temperature. Intranasal infection was performed with 25 μL PBS containing 4 × 10^10^ CFU *K. pneumoniae*. PBS alone was used as the vehicle control. At 48 h post-infection, mice were treated intraperitoneally with either 500 μL 50 mM valeric acid or 500 μL PBS. After 24 h of treatment, all animals were euthanized, and serum and lung tissues were harvested for further analysis.

### 2.4 Tissue sampling

After treatment, animals were anesthetized with isoflurane and blood was collected from the retroorbital sinus. Following euthanasia by cervical dislocation, the lungs were precisely dissected, weighed, immediately immersed in liquid nitrogen, and stored at −80 °C for subsequent analysis.

### 2.5 Histopathology and immunohistochemistry

Mouse lung tissue was fixed in 4% paraformaldehyde (PFA) for 24 h, paraffin-embedded, and slides were prepared (5 μm). Slides were hematoxylin and eosin (H&E) stained to assess tissue inflammation and injury. The severity of lung tissue injury was evaluated using the previously described method (modified Smith scoring system). Pathological assessment criteria included the degree of pulmonary edema, extent of alveolar and interstitial inflammatory cell infiltration, area of alveolar hemorrhage, incidence of atelectasis, and presence of hyaline membrane formation. Scoring criteria were as follows: 0 points indicated no pathological changes; 1 point indicated <25% lesion area; 2 points indicated 25%–50% lesion area; 3 points indicated 51%–75% lesion area; 4 points indicated >75% lesion area. Individual scores were summed to obtain a total injury score, ultimately expressed as a mean value.

For immunohistochemistry (IHC), after deparaffinization and rehydration, a citrate solution was used for antigen retrieval of lung tissue slides. After blocking with 10% goat serum, the primary antibody anti-IL-17A (Abclonal) was incubated overnight at 4 °C. The next day, slides were washed with PBS and incubated with HRP-labeled secondary antibody for 50 min at RT. Chromogenic reaction was performed with DAB, followed by counterstaining with hematoxylin. After dehydration and mounting, slides were imaged under a light microscope.

### 2.6 RNA sequencing and data analysis

Total RNA from mouse lung tissues was used to transcriptome sequencing. RNA integrity was assessed using an Agilent 5,400 Bioanalyzer (Agilent Technologies, United States). RNA purity and quantification were evaluated using a NanoDrop 2000 spectrophotometer (Thermo Fisher Scientific, United States). RNA libraries were prepared using the NEBNext Ultra RNA Library Prep Kit for Illumina (NEB, United States) following the manufacturer’s instructions and sequenced using Illumina HiSeq X Ten for 150-base paired-end reads. All reads were aligned to the mouse reference genome (GRCm39) using Hisat2. The DESeq2 was used to normalize raw counts and identify differentially expressed genes (DEGs). Volcano plots and heatmap were performed using the R package ggplot2 v3.4.4. Gene set enrichment analysis (GSEA) was performed using the R package clusterProfiler v4.8.3 to determine whether predefined gene sets were significantly enriched between the corresponding conditions.

### 2.7 Metabolomics preparations for serum samples

The serum sample was thawed on ice, and then 50 μL of serum sample was transferred to an EP tube. After the addition of 200 μL of extract solution (acetonitrile: methanol = 1:1, containing isotopically-labelled internal standard), the samples were vortexed for 30 s, sonicated for 10 min in ice-water bath, and incubated for 1 h at −40 °C to precipitate proteins. Then the sample was centrifuged at 12,000 rpm for 15 min at 4 °C. The resulting supernatant was transferred to a fresh glass vial for non-targeted metabolomics liquid chromatography-mass spectrometry (LC-MS) detection. The quality control (QC) sample was prepared by mixing an equal aliquot of the supernatants from all of the samples.

### 2.8 LC-MS analysis

LC-MS/MS analyses were performed using an UHPLC system (Vanquish, Thermo Fisher Scientific) with a UPLC BEH Amide column (2.1 mm × 100 mm, 1.7 μm) coupled to Q Exactive HFX mass spectrometer (Orbitrap MS, Thermo Fisher Scientific). The mobile phase consisted of 25 mmol/L ammonium acetate and 25 ammonia hydroxide in water (pH = 9.75) (A) and acetonitrile (B). The analysis was carried with elution gradient as follows: 0–0.5 min, 95% B; 0.5–7.0 min, 95%–65% B; 7.0–8.0 min, 65%–40% B; 8.0–9.0 min, 40% B; 9.0–9.1 min, 40%–95% B; 9.1–12.0 min, 95% B. The column temperature was 30 °C. The auto-sampler temperature was 4 °C, and the injection volume was 3 μL.

The QE HFX mass spectrometer was used for its ability to acquire MS/MS spectra on information-dependent acquisition mode in the control of the acquisition software (Xcalibur, Thermo). In this mode, the acquisition software continuously evaluates the full scan MS spectrum. The ESI source conditions were set as following: sheath gas flow rate as 50 Arb, Aux gas flow rate as 10 Arb, capillary temperature 320 °C, full MS resolution as 60,000, MS/MS resolution as 7,500, collision energy as 10/30/60 in NCE mode, spray Voltage as 3.5 kV (positive) or −3.2 kV (negative), respectively.

Raw data were converted to mzXML format using ProteoWizard and processed with an in-house R program based on XCMS for peak detection, alignment, and integration. Metabolite annotation was performed against an in-house MS2 database (BiotreeDB). Peaks with missing values (ion intensity = 0) in more than 50% of samples or 20% of QC samples, as well as isotopic ions, were removed. Signal drift was corrected using Robust Loess Signal Correction based on QC samples, with a relative standard deviation threshold of 30% applied to assess repeatability. After quality filtering, 3,863 features (positive mode) and 2,946 features (negative mode) were retained. The preprocessed data were normalized by the total ion intensity (sum normalization) for subsequent analyses.

### 2.9 Quantification of SCFA in serum

Serum samples were transferred into 1.5 mL EP tubes. Add 0.05 mL 50% H_2_SO_4_ and 0.2 mL of extracting solution (25 mg/L stock in methyl tert-butyl ether) as internal standard, vortex mixing for 30 s, oscillations in 10 min, then ultrasound treated for 10 min (incubated in ice water). Centrifuge for 15 min at 10,000 rpm, 4 °C. Keep at −20 °C for 30 min. Transfer the supernatant to a new 2 mL glass vial for SCFA analysis by gas chromatography-mass spectrometry (GC-2030-QP2020 NX, Shimadzu, Germany) fitted with a HP-FFAP capillary column (30 m × 0.25 mm inner diameter; Agilent Technologies, Germany).

### 2.10 Calu-3 cell line and air-liquid interface culture

The human bronchial epithelial cell line Calu-3 cells (ATCC, HTB-55) were cultured in MEM medium supplemented with 10% (v/v) fetal bovine serum (Gibco), 1% (v/v) penicillin-streptomycin (Gibco), 1% (v/v) L-glutamine (Gibco), and 1% (v/v) nonessential amino acids (Gibco). When confluence reached 80%–90%, cells were digested with 0.05% trypsin-EDTA (Gibco). A 0.5 mL cell suspension at a density of 5 × 10^5^ cells/well was seeded onto the apical compartment of a 12-well insert (0.4 μm pore size, Corning). 1 mL of medium was added to the basal compartment of the insert for culture. When the culture reached full confluence, removed the apical medium, leaving only the basal medium to form an air-liquid interface (ALI). Culture was continued for 14 days, with the basal medium replaced every 2 days.

In the Calu-3 cells ALI culture model, the apical compartment was infected with *K. pneumoniae* suspension (MOI = 10) for 4 h. Subsequently, the basal compartment was treated with medium containing HDAC inhibitors or Wnt signaling pathway inhibitors for 24 h. Inhibitors used in the experiment included the HDAC1 inhibitor valproic acid, the HDAC2 inhibitor santacruzamate A, the HDAC3 inhibitor RGFP966, and the Wnt pathway inhibitor Wnt-C59 (Zhang et al., 2024; Severson et al., 2025). All reagents were prepared according to the manufacturer’s instructions and diluted to the desired working concentrations.

### 2.11 Scanning electron microscopy

After Calu-3 cells were cultured at the ALI for 14 days, they were collected for scanning electron microscopy (SEM) imaging. Using a scalpel, the transwell membrane was excised along the edge of the culture chamber and placed in a clean cell culture dish. The membrane was washed three times with PBS. To obtain SEM images of cilia, cell samples were fixed with 2.5% (w/v) glutaraldehyde overnight at 4 °C. Subsequently, samples were washed three times (5 min each) with 0.1 M phosphate buffer (PB, pH 7.2) and post-fixed with 1% osmium tetroxide at RT for 2 h. After another three washes with 0.1 M PB (5 min each), samples were dehydrated through a graded ethanol series (30%, 50%, 70%, 85%, 95%, each for 15 min; 100% ethanol, 3 × 15 min). The dehydrated samples were subjected to critical point drying using a Leica CPD300. To increase contrast and conductivity, the sample surfaces were coated with a gold sputter-deposited film. Finally, samples were imaged with a SU-8010 SEM (Hitachi, Japan).

### 2.12 Immunofluorescence staining and confocal imaging

At the indicated time points, the apical and basolateral compartments of the sample were washed with PBS. Subsequently, samples were fixed by addition of 4% PFA solution to the apical and basal compartments for 30 min at RT. Samples were washed three times with PBS. The membranes were cut from the inserts and processed for Immunofluorescence (IF). For IF, cells were blocked with immunostaining blocking solution (Beyotime) for 1 h at RT. Samples were incubated overnight at 4 °C with primary antibodies diluted in primary antibody dilution buffer. Primary antibodies included mouse anti-acetylated-α-tubulin (Proteintech), rabbit anti-ZO-1 (Abmart), rabbit anti-E-cadherin (Abmart), and rabbit anti-FOXJ1 (Proteintech). The next day, samples were washed three times with PBS, incubated with secondary antibody protected from light for 1 h at RT, and washed three times with PBS. Nuclei were counterstained with Hoechst 33342 for 10 min in the dark, then washed with PBS. Finally, samples were mounted with an anti-fade mounting medium. Images were acquired using a Leica TCS SP8 STED confocal microscope and analyzed with Leica LAS AF Lite software.

### 2.13 RNA extraction and quantitative real-time PCR

Total RNA was extracted from cultured cells using TRIzol reagent (Invitrogen, United States) according to the manufacturer’s instructions. RNA concentration and purity were assessed, and equal amounts of RNA were used for subsequent analysis. Quantitative real-time PCR (RT-qPCR) was performed on a QuantStudio 7 Real-Time PCR System (Applied Biosystems, United States) using the HiScript II One Step SYBR Green RT-qPCR Kit (Vazyme, China). The primer sequences used in this experiment are provided in Supplementary Table S1. Relative gene expression was calculated using the 2^−ΔΔCT^ method, with GAPDH or β-actin serving as the housekeeping gene.

### 2.14 Statistical analysis

The data are presented as the mean ± standard errors of the mean (SEM). Statistical analyses were performed via GraphPad Prism 8.3. A statistically significant difference was determined by Student’s t-test for normally distributed data or the Mann-Whitney test for non-normally distributed data. For comparisons involving three or more groups, one-way ANOVA was applied, followed by *post hoc* analysis via Tukey’s test for all pairwise comparisons or Dunnett’s test when multiple groups were compared against the control group. *p*-values below 0.05 are considered significant (*p < 0.05, **p < 0.01, ***p < 0.001, ****p < 0.0001).

## 3 Results

### 3.1 Serum metabolome reveals valeric acid associated with improved prognosis

**FIGURE 1:**
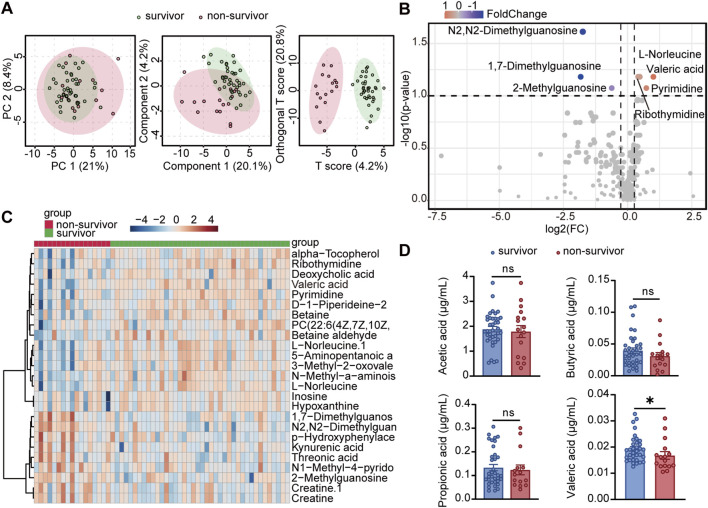
Serum metabolome reveals valeric acid associated with improved prognosis. **(A)** Principal component analysis on the serum metabolome profiles of survivors and non-survivors. **(B)** Volcano plot of differential metabolites between survivors and non-survivors. **(C)** Heatmap of serum metabolites between survivors and non-survivors, showing differentially expressed metabolites (arranged by relative abundance). **(D)** SCFA levels in serum samples from survivors and non-survivors detected by targeted metabolomics. ns, not significant; *p < 0.05 compared using Mann-Whitney test **(D)**.

We collected 57 serum samples from ICU patients with severe pneumonia. Serum collection was performed at the same time with bronchoalveolar lavage fluid (BALF) or sputum samples and within 1 day of admission. The 57 patients were divided into the survivor (40) and non-survivor (17) groups according to clinical outcomes, and their serum samples were analyzed by untargeted metabolomics. In terms of age, gender, and hospital length of stay, the two groups did not differ significantly (Supplementary Table S2). Our analysis however showed that the metabolomic profiles were distinguished more significantly between the survivor and non-survivor groups (Figure 1A). Among the differential metabolites, valeric acid was significantly enriched in the survivor group compared with the non-survivor group (Figures 1B,C; Supplementary Figure S1). Subsequently, targeted metabolomics analysis was performed to specifically quantify SCFA, confirming that valeric acid was significantly enriched in the survivor group (Figure 1D). These results suggest that valeric acid plays a potentially important role in the prognosis of severe pneumonia. Studies have indicated that SCFA (including valeric acid) are primarily produced by gut microbiota through the fermentation of indigestible carbohydrates, such as dietary fiber, and then absorbed into the bloodstream.

### 3.2 Valeric acid alleviates severity in bacterial infection of mouse lungs

**FIGURE 2:**
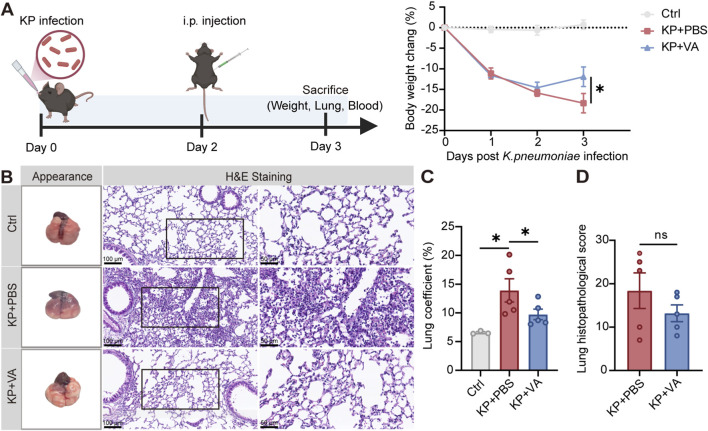
Valeric acid alleviates severity in bacterial infection of mouse lungs. **(A)** Experimental schematic of *K. pneumoniae*-infected mice treated with PBS or valeric acid, and the corresponding changes in body weight. **(B–D)** Evaluation of mouse lung morphology and histopathological changes **(B)**, lung coefficient **(C)**, and lung injury **(D)**. KP, *K. pneumoniae*; VA, valeric acid; Ctrl, uninfected control; KP + PBS, *K. pneumoniae*-infected mice treated with PBS; KP + VA, *K. pneumoniae*-infected mice treated with valeric acid. n = 3-5 mice per group. ns, not significant; *p < 0.05 compared using two-way ANOVA with Sidak’s multiple comparisons test **(A)**, unpaired Student’s t*-*test **(C)**, and Mann-Whitney test **(D)**.

To investigate the role of the differential metabolite valeric acid in the prognosis of severe pneumonia, given that *K. pneumoniae* is the most common bacterial pathogen causing both CAP and HAP (Russo and Marr, 2019; Juan et al., 2020; Grosjean et al., 2024; Algethamy et al., 2025), a mouse pneumonia model was established by intranasal inoculation with *K. pneumoniae*. Valeric acid (500 μL, 50 mM) or an equal volume of PBS was administered by i. p. injection at 48 h post-infection (Figure 2A; Supplementary Figure S2). Valeric acid treatment significantly improved body weight in *K. pneumoniae*-infected mice. Histopathological evaluation of lung lesions using H&E-stained tissue samples revealed reduced inflammation and cellular damage in the valeric acid group compared to the PBS group (Figure 2B). In addition, lung inflammation assessed by lung coefficients was significantly reduced in the valeric acid group compared with the PBS group (Figure 2C), further supporting the anti-inflammatory effect of valeric acid. Lung injury was assessed using the Smith scoring system (including five indexes of pulmonary edema, alveolar and interstitial inflammatory cell infiltration, alveolar and interstitial hemorrhage, pulmonary atelectasis, and hyaline membrane formation). The scores were lower in the valeric acid group compared with the PBS group, but the difference was not statistically significant (Figure 2D). Meanwhile, bacterial load in lung tissue was also lower in the valeric acid group than in the PBS group (Supplementary Figure S3). Taken together, these results suggest a potential protective effect of valeric acid against lung infections.

### 3.3 Valeric acid reduces IL-17 inflammation and upregulates cilia-related pathways

**FIGURE 3:**
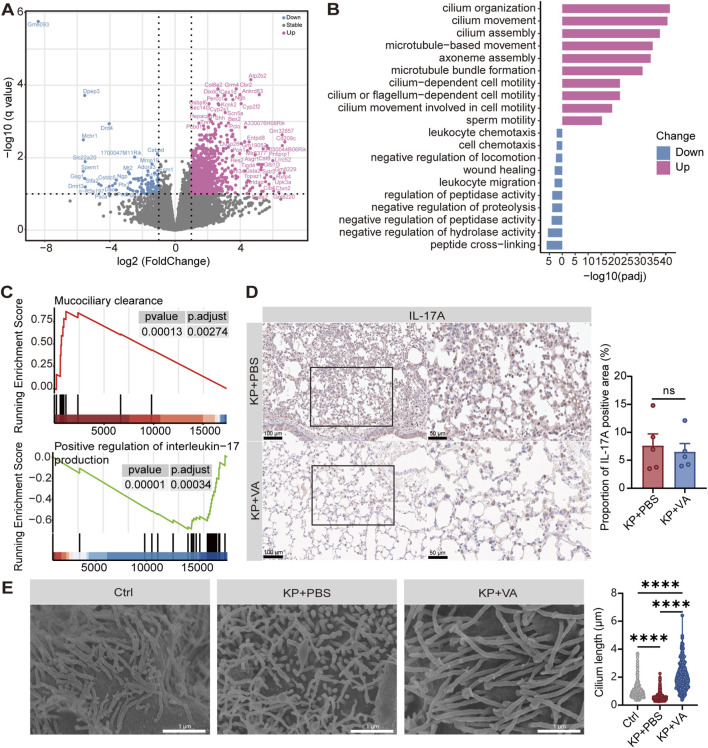
Valeric acid reduces IL-17 inflammation and upregulates cilia-related pathways. **(A)** Volcano plot of DEGs (log2 fold change ≥1, -log(P) ≥ 1) in *K. pneumoniae*-infected mice treated with PBS or valeric acid. Red and blue dots represent significantly upregulated and downregulated genes, respectively. **(B)** Top 10 enriched terms of the genes by Gene Ontology biological processes (GO-BP). **(C)** GSEA of DEGs in *K. pneumoniae*-infected mice treated with PBS or valeric acid. **(D)** Representative IL-17A staining images and scoring of lung tissues. **(E)** Representative SEM images of Calu-3 cells cultured at ALI after treatment and the quantification of cilium length. Ctrl, uninfected control; KP + PBS, *K. pneumoniae*-infected mice treated with PBS; KP + VA, *K. pneumoniae*-infected mice treated with valeric acid. n = 5 mice per group. ns, not significant; ****p < 0.0001 compared using unpaired Student’s t*-*test **(D)** and one-way ANOVA with Tukey’s multiple comparisons test **(E)**.

To further explore the potential mechanisms of valeric acid in the prognosis of severe pneumonia, we performed RNA-seq analysis on lung tissues from *K. pneumoniae*-infected mice treated with or without valeric acid. RNA-seq analysis identified 100 significantly upregulated and 917 significantly downregulated genes in the valeric acid group compared with the PBS group (Figure 3A). Pathway analysis further demonstrated that cilia-related pathways were markedly upregulated in the valeric acid group (Figure 3B). In addition, RNA-seq analysis of lung tissues from uninfected mice further indicated that valeric acid promotes pulmonary epithelial cilia assembly only following infection-induced injury (Supplementary Figures S4-S6). Moreover, GSEA confirmed that the valeric acid group upregulated the mucociliary clearance (MCC) pathway while downregulating the IL-17 signaling pathway (Figure 3C). Given that the valeric acid-FFAR2 (free fatty acid receptor 2)-IL-17 axis has been previously reported (Zeng et al., 2023), IL-17A expression was further examined in lung tissues from *K. pneumoniae*-infected mice with or without valeric acid treatment by IHC. The expression of IL-17A showed a decreasing trend in the valeric acid group compared with the PBS group (Figure 3D).

In addition, we validated our findings using an *in vitro* Calu-3 cell ALI culture model, which promotes ciliary differentiation (Supplementary Figures S7,S8). SEM revealed that the valeric acid group significantly improved cilia length (Figure 3E), which potentially enhanced the pathogen clearance capacity. RNA-seq and GSEA analyses revealed that valeric acid treatment upregulated pathways associated with cilia assembly and MCC, accompanied by improved cilia length in infected lung tissues. Collectively, these findings suggest that valeric acid plays a protective role in lung infection by reducing IL-17-associated inflammation and further identify a novel role of valeric acid in regulating ciliary function.

### 3.4 Valeric acid upregulates cilia assembly-related genes

**FIGURE 4:**
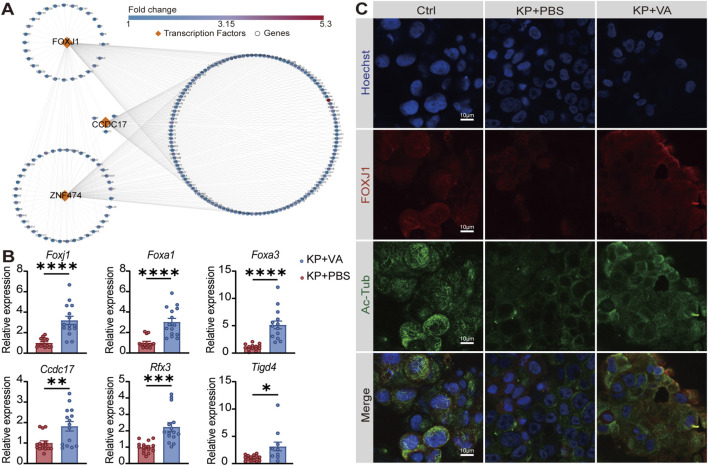
Valeric acid upregulates cilia assembly-related genes. **(A)** TFs-gene regulatory network of DEGs in *K. pneumoniae*-infected mice treated with PBS or valeric acid. Orange diamonds denote predicted key TFs; blue circles denote putative target genes; edges indicate predicted regulatory relationships. **(B)** mRNA expression levels of cilia-associated TFs in *K. pneumoniae*-infected mice treated with PBS or valeric acid. **(C)** Representative IF staining images of Calu-3 cells cultured at ALI after treatment. Nuclei are labeled with Hoechst (blue), and ciliary markers include FOXJ1 (red) and acetylated α-tubulin (green). Ac-Tub, acetylated α-tubulin. **(B)** 3 independent replicates with 5 mice per group in each replicate. **(C)** 3 independent experiments. *p < 0.05, **p < 0.01, ***p < 0.001, ****p < 0.0001 compared using Mann-Whitney test **(B)**.

To identify the key transcriptional regulators and upstream signaling mechanisms driving this program, we combined *in vitro* ALI culture model with *in vivo* qPCR validation. Using the ChEA3 database for transcription factor (TF) enrichment analysis, we predicted Foxj1, Ccdc17, and Znf474 as pivotal regulators mediating the valeric acid-induced activation of cilia-related pathways in mouse lungs. The constructed transcriptional regulatory network further showed that the genes connected to these TFs were predominantly involved in cilia assembly (Figure 4A). Foxj1 is a master TF essential for ciliary differentiation and maintenance (Li et al., 2023). Consistently, *in vivo* qPCR analysis demonstrated that *Foxj1* and its co-regulatory partners—such as *Foxa1*, *Foxa3*, *Rfx3*, *Ccdc17*, and *Tigd4*—were collectively upregulated following valeric acid treatment in mouse lung tissues (Figure 4B). Meanwhile, in the *K. pneumoniae*-infected Calu-3 cell ALI culture model, IF staining showed that valeric acid treatment restored and enhanced both FOXJ1 and ciliary structures marked by acetylated α-tubulin (Figure 4C), suggesting that valeric acid reactivates the ciliary differentiation program under infectious stress.

### 3.5 Valeric acid inhibits HDAC activities upstream of Wnt pathway

**FIGURE 5:**
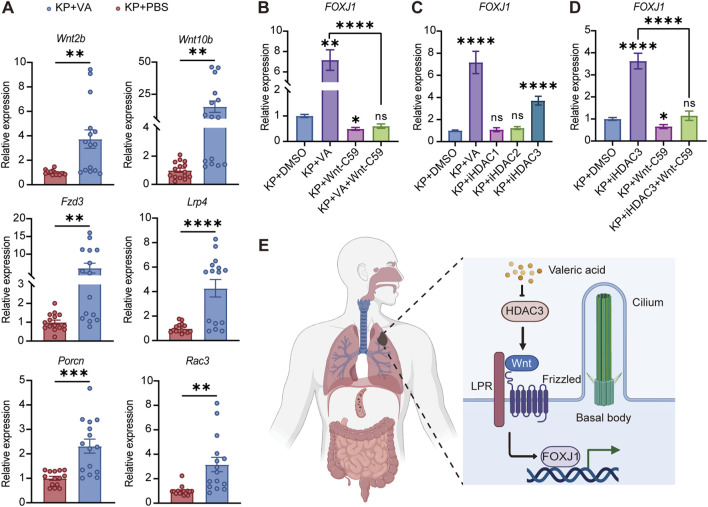
Valeric acid inhibits HDAC activities upstream of Wnt pathway. **(A)** mRNA expression levels of Wnt signaling pathway-related genes in *K. pneumoniae*-infected mice treated with PBS or valeric acid. **(B–D)** mRNA expression levels of Calu-3 cells cultured at ALI treated with valeric acid, HDAC inhibitors, or the Wnt inhibitor (Wnt-C59). **(E)** Overview schematic of the study (created by Biorender.com). KP + PBS, *K. pneumoniae*-infected mice treated with PBS; KP + VA, *K. pneumoniae*-infected mice treated with valeric acid; VA, valeric acid; iHADC1, HDAC1 inhibitor; iHDAC2, HDAC2 inhibitor; iHDAC3, HDAC3 inhibitor. **(A)** 3 independent replicates with 5 mice per group in each replicate. **(B–D)** n = 3, 3 independent experiments. ns, not significant; *p < 0.05, **p < 0.01, ***p < 0.001, ****p < 0.0001 compared using Mann-Whitney test **(A)** and Kruskal-Wallis test with Dunn’s multiple comparisons test **(B–D)**.

Previous studies have demonstrated that the Wnt signaling pathway serves as a key upstream mechanism regulating FOXJ1 expression (Caron et al., 2012; Walentek et al., 2012; Miyatake et al., 2015; Zhu et al., 2015; Zhang et al., 2020; Garcia-Alonso et al., 2021; Seidl et al., 2023). In *K. pneumoniae*-infected mouse lung tissues, valeric acid treatment markedly increased the transcriptional levels of key components and co-factors of the Wnt/β-catenin pathway, including *Wnt2b*, *Wnt10b*, *Porcn*, *Fzd3*, *Lrp4*, and *Rac3* (Figure 5A), indicating a broad activation of Wnt signaling. Consistently, *in vitro* experiments showed that inhibition of Wnt signaling with Wnt-C59 not only reduced FOXJ1 expression but also abolished the valeric acid-induced upregulation of FOXJ1 (Figure 5B). Collectively, these results suggested that valeric acid promotes cilia assembly and maintenance by activating the Wnt signaling pathway to enhance FOXJ1 expression, highlighting the Wnt-FOXJ1 axis as a key pathway mediating the cilia-promoting effects of valeric acid.

Given that valeric acid has been reported to suppress HDAC activity (Shi et al., 2021), we further explored the mechanism by which valeric acid activates the Wnt-FOXJ1 axis through selective inhibition of HDAC activity. Under *K. pneumoniae* infection, specific inhibition of HDAC3 significantly increased FOXJ1 expression, mimicking the effects of valeric acid treatment, whereas inhibition of HDAC1 or HDAC2 showed no significant effect (Figure 5C). These findings indicate that inhibition of HDAC3 activity recapitulates the valeric acid-induced upregulation of FOXJ1, suggesting that HDAC3 is a key inhibitory node in this regulatory pathway. Furthermore, blockade of Wnt signaling with Wnt-C59 eliminated the FOXJ1 upregulation triggered by HDAC3 inhibition (Figure 5D), and similarly abrogated the valeric acid-induced increase in FOXJ1 (Figure 5B). Together, these results establish a causal relationship within the HDAC-Wnt-FOXJ1 axis, indicating that HDAC inhibition acts as an upstream event leading to Wnt activation.

## 4 Discussion

Here, to address the high mortality of severe pneumonia and the lack of effective host-directed interventions, we identified valeric acid as a metabolite significantly associated with favorable clinical outcomes and showed that it attenuated pulmonary inflammation and tissue injury in bacterial infection models. We further found that, under infectious stress, valeric acid upregulated pathways involved in cilia assembly and MCC, thereby improving ciliary function. Mechanistically, valeric acid promoted ciliogenesis through an HDAC3-Wnt-FOXJ1 axis. Our study indicated that valeric acid exerted a protective effect on the prognosis of severe pneumonia.

Airway epithelial cilia constituted the first physical barrier of the innate immune system in the respiratory tract (Kuek and Lee, 2020). Through coordinated ciliary beating, they drove the MCC system to effectively clear inhaled pathogens and harmful particles, thereby maintaining airway homeostasis and reducing the risk of infection (Fliegauf et al., 2013; Kuek and Lee, 2020; Laborie et al., 2025). However, in conventional studies on pneumonia and related respiratory diseases, cilia were often regarded merely as passive mechanical structures for clearance, while their dynamic regulatory mechanisms and functional roles in disease development remain largely underappreciated (Yaghi and Dolovich, 2016; Kuek and Lee, 2020). Previous studies have shown that multiple respiratory viruses downregulated genes required for ciliogenesis and could induce dedifferentiation of ciliated cells (Wu et al., 2016; Robinot et al., 2021; Ma et al., 2022); ciliary dysfunction was tightly linked to chronic airway diseases such as chronic obstructive pulmonary disease and asthma (Thomas et al., 2010; Yaghi and Dolovich, 2016; Khelloufi et al., 2018). Consistent with this, our multifaceted evidence indicates that impaired ciliary function is an important driver of severe pneumonia progression, which activation of FOXJ1 and its upstream pathways can restore MCC-mediated defense.

SCFA, the principal metabolites generated by gut microbial fermentation of dietary fiber, have anti-inflammatory and immunomodulatory effects (Sivaprakasam et al., 2016; Visekruna and Luu, 2021). Valeric acid has been reported to suppress Th17 cell differentiation and to exhibit antitumor effects (Luu et al., 2019; 2021; Lau et al., 2024). In our study, valeric acid inhibited IL-17 biosynthesis-related signaling while upregulating MCC pathways. In our *in vivo* experiments, valeric acid treatment markedly reduced lung inflammation and injury, suggesting a broad protective action against infection-induced pulmonary damage, which likely related to the outcomes observed in our severe pneumonia ICU cohort. Notably, we demonstrated that HDAC3 inhibition leads to higher levels of histone acetylation, consequently structural changes to chromatin and thus transcriptional regulation of genes in the Wnt pathway. This activation then subsequently upregulates FOXJ1 expression and eventually cilia assembly. Together, these findings establish an HDAC3-Wnt-FOXJ1 signaling cascade. To our knowledge, this cilia-centric regulatory mechanism has not been reported previously.

These findings provide promising new translational research directions for the prevention and treatment of severe pneumonia. Building on the gut-lung axis, novel strategies targeting the gut microbiota or its metabolites are emerging for the management of lung disease. Valeric acid, as a key metabolite, provides multiple potential therapeutic approaches. First, given the association between serum valeric acid and clinical outcomes in our cohort, valeric acid could be incorporated into a clinical metabolomics-based stratification framework for early risk identification and dynamic assessment of therapeutic response. Second, exogenous valeric acid supplementation, dietary fiber interventions to enhance endogenous production, and the use of HDAC3 inhibitors or small molecules targeting the Wnt pathway could serve as effective approaches to reinforce mucociliary defenses. Nonetheless, our study has limitations. Animal models cannot fully recapitulate the heterogeneity of human disease, and our sample size was modest; larger clinical cohorts are needed to validate the association between valeric acid and prognosis. Although we delineated the HDAC3-Wnt-FOXJ1 axis, causal inference would be strengthened by genetic manipulations (e.g., airway epithelial HDAC3 deletion and FOXJ1 inhibition) and quantitative MCC readouts (e.g., ciliary beat frequency and particle-tracking clearance).

In summary, we showed that higher serum valeric acid levels were positively associated with prognosis in ICU patients with severe pneumonia, and we validated the protective effects of valeric acid against lung inflammation and tissue damage in infection models. Mechanistically, valeric acid enhanced ciliary biogenesis and function by inhibiting HDAC3, activating Wnt signaling, and upregulating FOXJ1, which ultimately improved clinical outcomes in severe pneumonia. Our work revealed a previously unappreciated regulatory pathway linking the microbial metabolite valeric acid to host epithelial ciliary defense. This discovery not only deepens our understanding of the gut-lung axis but also provided a theoretical basis for interventions against severe pneumonia and related respiratory diseases.

## Data Availability

Data are available in manuscript or Supplementary Materials. Meta - transcriptomic sequencing data are deposited to National Microbiological Data Center under the link: https://nmdc.cn/resource/genomics/project/detail/NMDC10018476.
