# Supplementary material for: Serum valeric acid stimulates lung epithelial cilia assembly and improves prognosis in patients with severe respiratory infections
Source: Front Pharmacol. 2026 Jan 12;16:1761517. doi: 10.3389/fphar.2025.1761517 (PMC12832812; doi:10.3389/fphar.2025.1761517)
Supplement: Supplementary file 1 [file DataSheet1.docx]

Supplementary Material

# Supplementary Figures and Tables

## Supplementary Figures


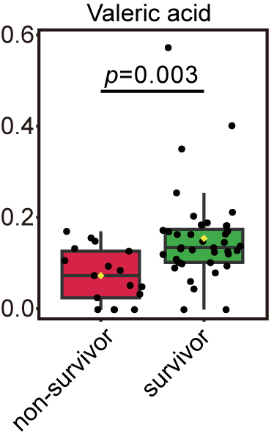


**Supplementary Figure 1.** **Expression levels of valeric acid in survivor and non-survivor groups as determined by serum untargeted metabolomics analysis.** The red box represents the non-survivor group, and the green box represents the survivor group. Statistical analysis was performed using Mann-Whitney test.

**
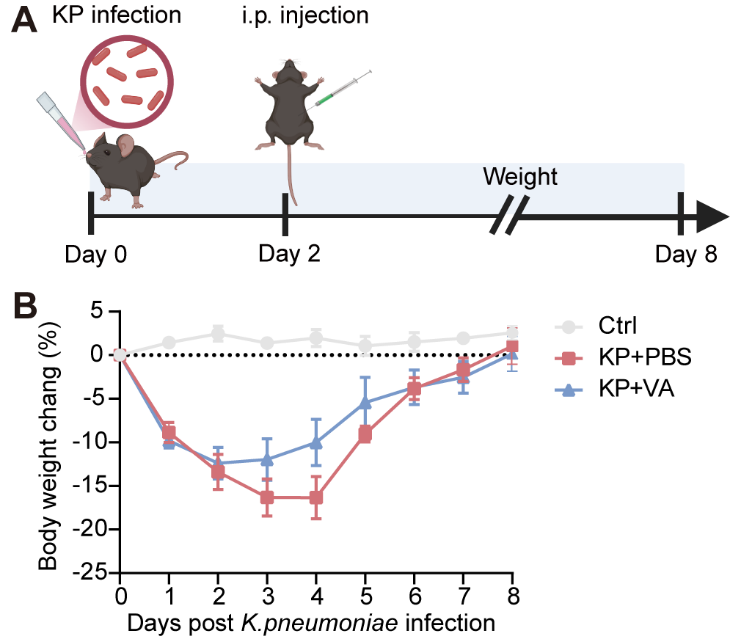
**

**Supplementary Figure 2. Effects of valeric acid treatment on body weight changes in mice infected with *K. pneumoniae*.** (A-B) Experimental schematic of *K. pneumoniae*-infected mice treated with PBS or valeric acid, and the corresponding changes in body weight. Mice were infected with *K. pneumoniae* on day 0, followed by i.p. injection of PBS or valeric acid on day 2. Body weight was monitored from day 0 to day 8. Data are presented as mean ± SEM. Ctrl, uninfected control; KP+PBS, *K. pneumoniae*-infected mice treated with PBS; KP+VA, *K. pneumoniae*-infected mice treated with valeric acid. n = 5 mice per group.

**
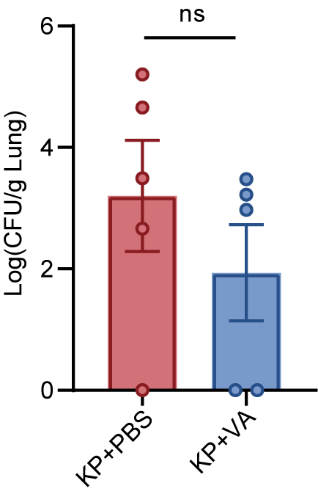
**

**Supplementary Figure 3. Pulmonary bacterial burden (CFU/g lung tissue) in *K. pneumoniae*-infected mice treated with PBS or valeric acid.** Data are presented as mean ± SEM. n = 5 mice per group. Statistical analysis was performed using Mann-Whitney test. ns, not significant.

**
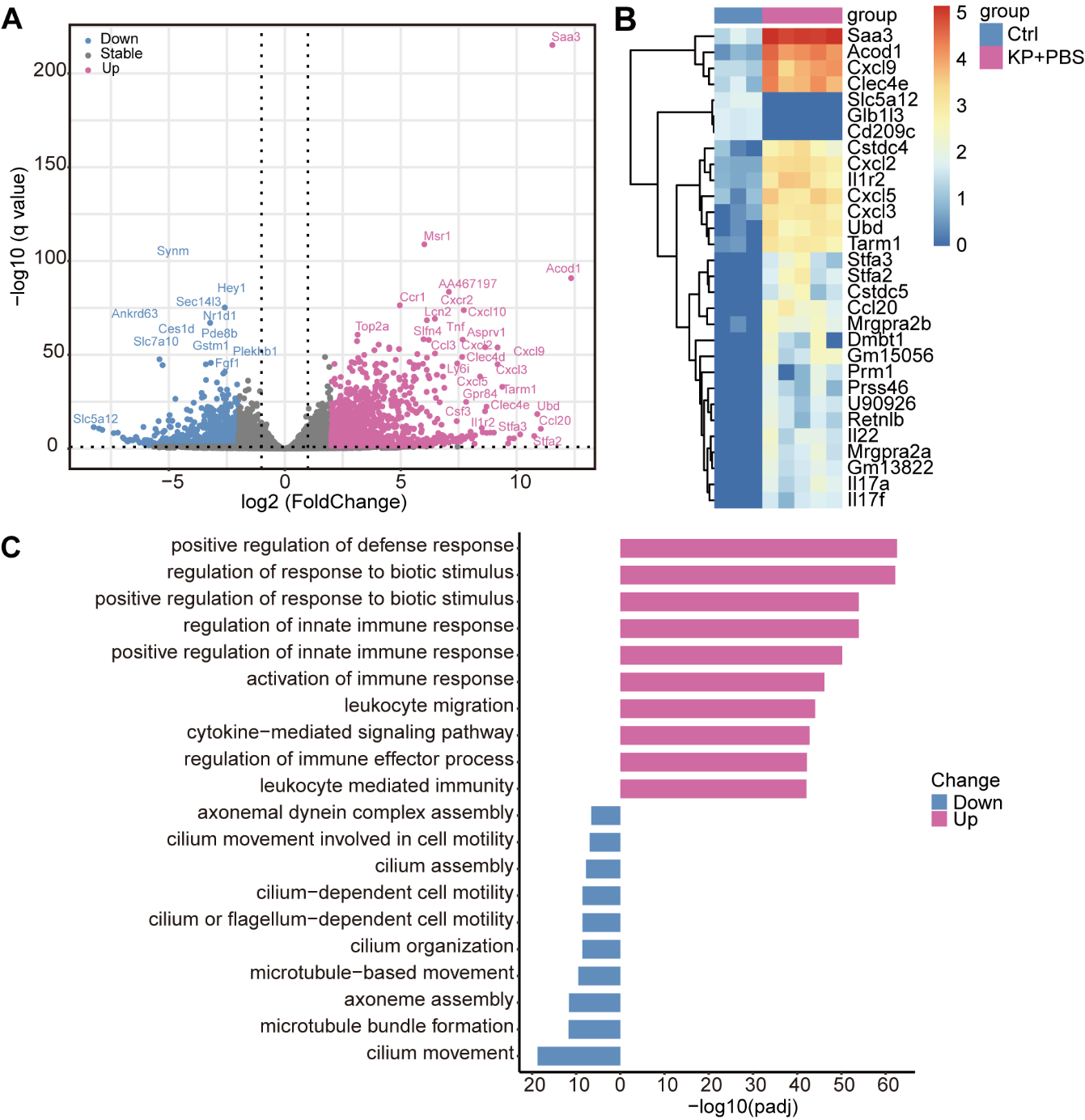
**

**Supplementary Figure 4. Differential Gene Expression Analysis in mice lung tissue.** (A) Volcano plot comparing DEGs in uninfected control mice and *K. pneumoniae*-infected mice treated with PBS. 1191 genes were up-regulated and 937 genes were down-regulated in the *K. pneumoniae*-infected mice treated with PBS (log2 fold change > 2, -log(P) > 1.3). (B) Heatmap comparing the expression levels of differentially expressed genes between the uninfected control mice and *K. pneumoniae*-infected mice treated with PBS. (C) GO enrichment analysis of the differentially expressed genes identified in (A). Ctrl, uninfected control; KP+PBS, *K. pneumoniae*-infected mice treated with PBS.

**
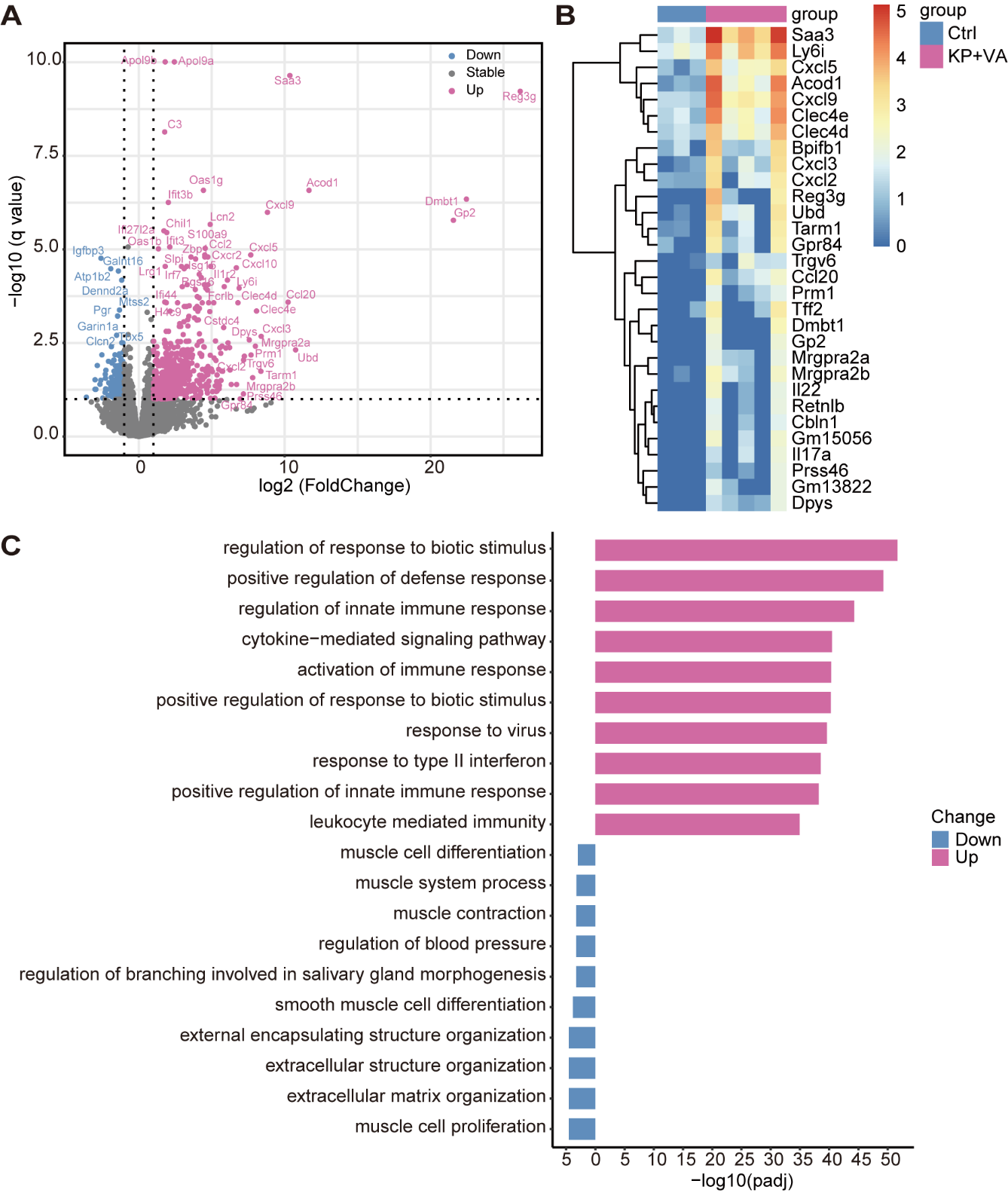
**

**Supplementary Figure 5. Differential Gene Expression Analysis in mice lung tissue.** (A) Volcano plot comparing DEGs in uninfected control mice and *K. pneumoniae*-infected mice treated with valeric acid. 712 genes were up-regulated and 191 genes were down-regulated in the *K. pneumoniae*-infected mice treated with valeric acid group (log2 fold change > 1, -log(P) > 1). (B) Heatmap comparing the expression levels of differentially expressed genes between the uninfected control mice and *K. pneumoniae*-infected mice treated with valeric acid. (C) GO enrichment analysis of the differentially expressed genes identified in (A). Ctrl, uninfected control; KP+VA, *K. pneumoniae*-infected mice treated with valeric acid.

**
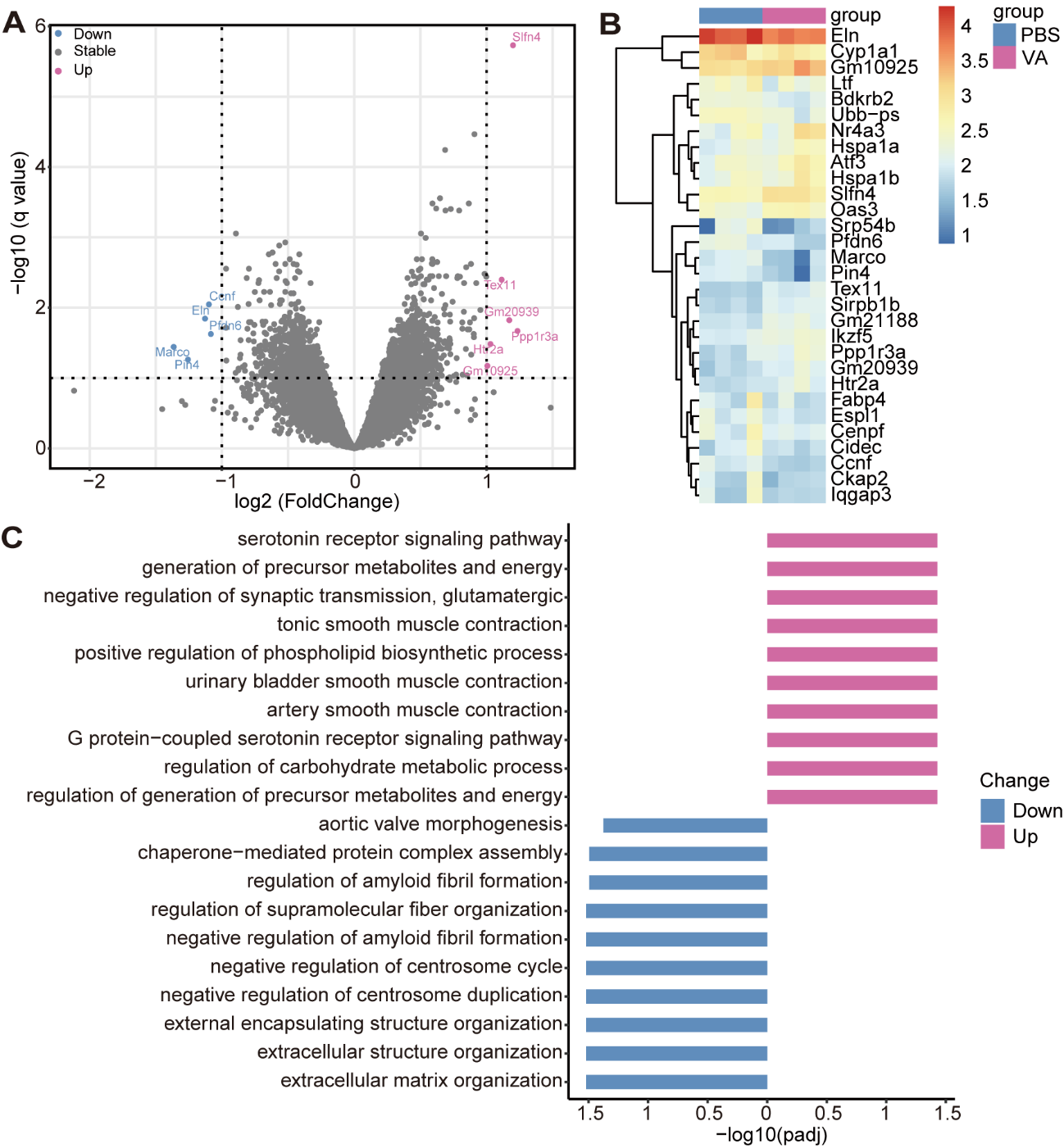
**

**Supplementary Figure 6. Differential Gene Expression Analysis in mice lung tissue.** (A) Volcano plot comparing DEGs in uninfected mice treated with PBS or valeric acid. 6 genes were up-regulated and 5 genes were down-regulated in the valeric acid group (log2 fold change > 1, -log(P) > 1). (B) Heatmap comparing the expression levels of differentially expressed genes between the uninfected mice treated with PBS or valeric acid. (C) GO enrichment analysis of DEGs identified in (A). VA, valeric acid.

**
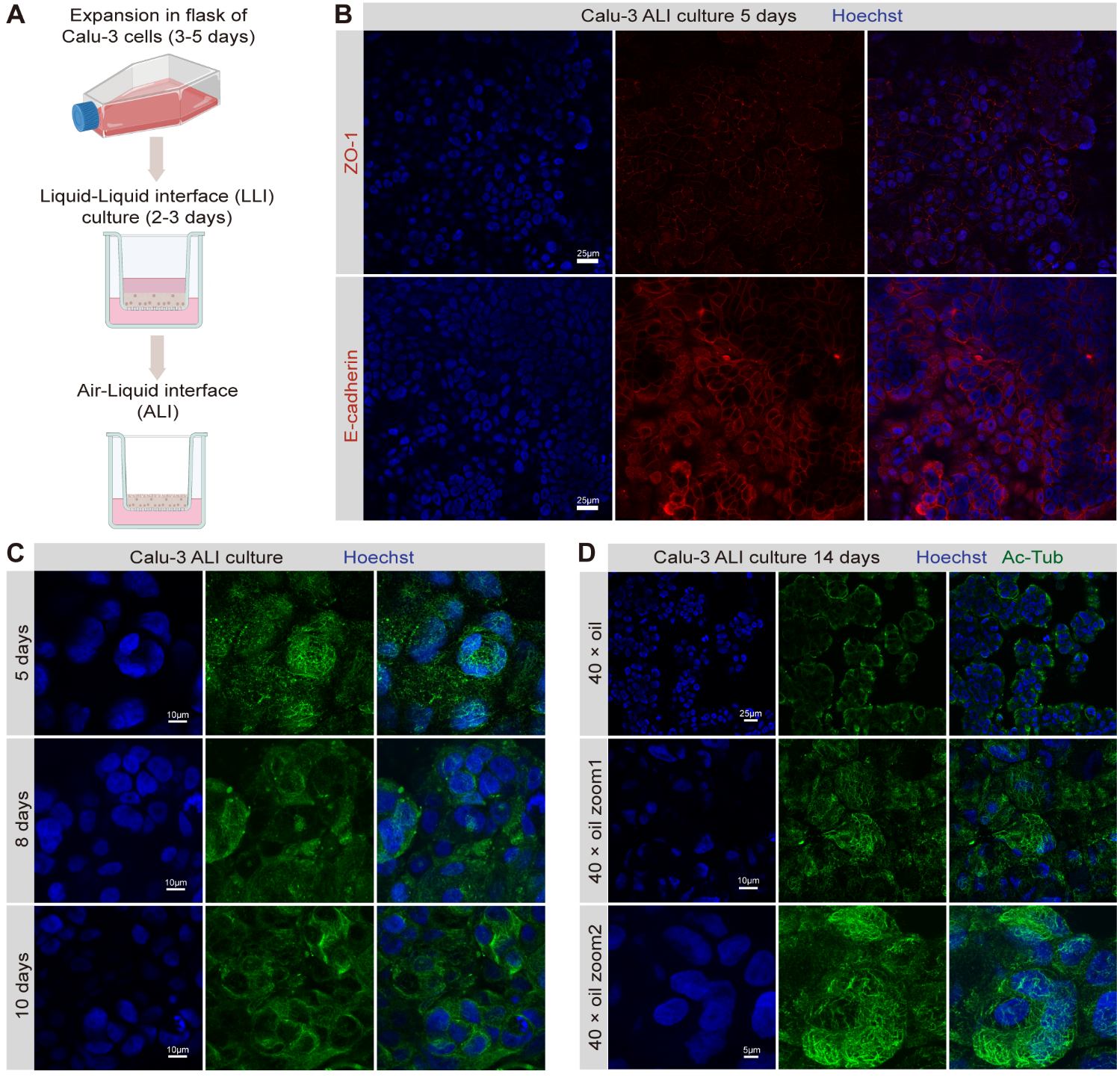
**

**Supplementary Figure 7. Calu-3 cell ALI culture and ciliary differentiation.** (A) Schematics of Calu-3 cells culture process to develop fully differentiated (14 days) on transwell inserts. (B) IF staining of ZO-1 and E-cadherin in Calu-3 cells. Calu-3 cells were maintained at ALI culture condition for 5 days. Tight junction formation and adherens junctions were evaluated by staining the cells with ZO-1 and E-cadherin antibodies, respectively. (C-D) Development of ciliary differentiation in Calu-3 cells cultured at ALI (0 to 14 days) was visualized by IF. Ac-Tub, acetylated-α-tubulin. (B-D) 3 independent experiments.

**
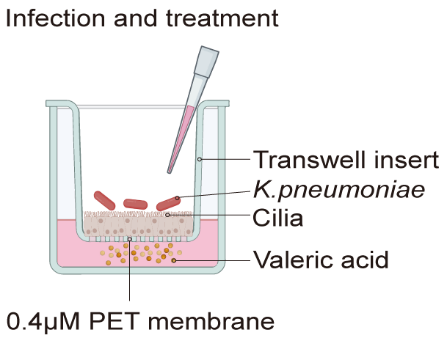
**

**Supplementary Figure 8. Schematic of the apical infection and the basolateral treatment of fully differentiated Calu-3 ALI cultured on Transwell inserts.** Calu-3 cells were maintained at ALI for 14 days within a transwell insert featuring a 0.4 μm PET membrane. *K. pneumoniae* was inoculated at the apical compartment, while valeric acid was add to the basolateral compartment.

## Supplementary Tables

# Supplementary Table 1. Sequences of primers used in this study

| For human: |  |  |
| --- | --- | --- |
| **Gene name** | **Forward sequence (5' to 3')** | **Reverse sequence (5' to 3')** |
| FOXJ1 | CCTGTCGGCCATCTACAAGT | CGGATTGAATTCTGCCAGGT |
| GAPDH | GGAGCGAGATCCCTCCAAAAT | GGCTGTTGTCATACTTCTCATGG |
|  |  |  |
| For mouse: |  |  |
| **Gene name** | **Forward sequence (5' to 3')** | **Reverse sequence (5' to 3')** |
| Foxj1 | CCACCCTACTCCTATGCCACTCTC | ATGGCGGAAGTAGCAGAAGTTGTC |
| Foxa1 | ATGAGAGCAACGACTGGAACA | TCATGGAGTTCATAGAGCCCA |
| Foxa3 | TACTACCGGGAGAACCAGCA | TCCAGGCCGCTGAAATAAGG |
| Ccdc17 | ATTCTGTTACCTGCCCCTGC | GCTGGAGGTAATGCTGTGGT |
| Rfx3 | CTCCAGCTGTCTCAGAGATTTT | CACCTGAGCTGGGTAGACAT |
| Tigd4 | GCCCAGAAACGAAGAGTGGT | TCTGGGTGGCTTCACAAGTC |
| Wnt2b | GCACGGCTGTTCGGAGATTC | TGCGTCGGAAGTCTGAGAGTG |
| Wnt10b | TGGGACGCCAGGTGGTAA | CTGACGTTCCATGGCATTTG |
| Fzd3 | GAAGCAAAGCAGGGAGTGTC | ATGCTGCCGTGAGGTAGTCT |
| Lrp4 | GTGTGGCAGAACCTTGACAGTC | TACGGTCTGAGCCATCCATTCC |
| Porcn | TTCAGCCGCCAGGAATTTTTC | GGTAAGACGGTAACCCCAGC |
| Rac3 | CCCACACACACCCATCCTTC | TGGAGCTATATCCCAGAAAAAGGAG |
| β-actin | GCAGGAGTACGATGAGTCCG | ACGCAGCTCAGTAACAGTCC |

**Supplementary Table 2. Clinical characteristics of ICU patients in this study**

|  | | | | |
| --- | --- | --- | --- | --- |
| **Characteristic** | **Overall (n=57)** | **Survivor (40)** | **Non-survivor (17)** | ***p* Value** |
| Age yr | 75 (61.5-86.5) | 79.5 (59.5-85.3) | 72 (70-88) | 0.64 |
| Sex no. (%) |  |  |  | 0.52 |
| Male | 41 (71.9) | 30 (75) | 11 (64.7) |  |
| Female | 16 (28.1) | 10 (25) | 6 (35.2) |  |
| Hospital length of stay, d | 14 (10-24) | 15 (11-23) | 9 (6-24) | 0.11 |
| Values are presented as median (interquartile range, IQR) for continuous variables and as number (percentage) for categorical variables. *p* values were calculated using the Mann-Whitney U test for continuous variables and Fisher’s exact test for categorical variables, as appropriate. yr, year; d, day. | | | | |
